# Iowa’s Need

**Published:** 1900-02

**Authors:** 


### IOWA'S NEED.

THE bill to regulate the practice of veterinary science in Iowa has passed the lower branch of the legislature by an overwhelming majority, and is now in the Senate. It will need in the upper body the strong arm of support of every active veterinarian in the State. Its provisions are liberal, its demands conservative, and its promises salutary to the best interests of the people of that State, and a safeguard in the future to much saving to the people —a properly educated body of veterinarians. It should have gone farther in encouragement of the State University's Veterinary Department by designating a definite time when all future graduates shall have complied with the advanced curriculum of to-day in most all of our veterinary colleges and that laid down by the American Veterinary Association, as the standard of requirements for her members. Iowa needs such a law, the profession will advance more rapidly under its fostering care, and its successful placing on the statutes will depend much upon a united support and energetic action upon the part of the profession.

